# Asynchronous Cross-Modal Dynamic Graph Learning for Intelligent Sensing of AI Computing Infrastructure Expansion

**DOI:** 10.3390/s26154902

**Published:** 2026-08-03

**Authors:** Zhe Xiang, Shangshan Chen, Xinrui Hu, Xu Xu, Yang Yang, Jingyi Yang, Yan Zhan

**Affiliations:** 1 Peking University, Beijing 100871, China; 2 China Agricultural University, Beijing 100083, China

**Keywords:** artificial intelligence-driven sensing, AI computing infrastructure, multimodal industrial sensing, asynchronous cross-modal alignment, dynamic heterogeneous graph

## Abstract

The rapid expansion of artificial intelligence computing infrastructure and the semiconductor industry has made the dynamic sensing of policy planning, technological innovation, capital investment, and physical construction essential for industrial monitoring and resource allocation. However, policy documents, remote-sensing imagery, patent relations, and economic indicators exhibit substantial heterogeneity, temporal misalignment, and variations in data quality, making it difficult for conventional unimodal or synchronously fused methods to accurately characterize the continuous evolution of construction activities. To address these challenges, a multimodal industrial sensing dataset for artificial intelligence computing infrastructure was constructed, and an artificial intelligence-driven asynchronous cross-modal dynamic graph prediction framework was developed. Differential temporal lags among policy announcements, capital investment, patent growth, and physical construction are learned through an asynchronous cross-modal alignment module. Interference caused by cloud contamination, missing text, patent disclosure delays, and incomplete economic observations is dynamically suppressed through a reliability-aware fusion mechanism. Time-varying propagation relationships among countries, enterprises, patents, and industrial parks are further modeled using an economically modulated dynamic heterogeneous graph. Experimental results demonstrate that, in next-window construction event prediction, the proposed model achieved an Accuracy of 91.72%, a Precision of 91.08%, a Recall of 90.64%, an F1-score of 90.86%, and a ROC-AUC of 95.67%, significantly outperforming baseline methods including XLM-R, Swin Transformer, TimesNet, HGT, and TGN. In construction intensity forecasting, the model achieved an MAE of 0.104, an RMSE of 0.153, a MAPE of 10.91%, an $R^{2}$ of 0.854, and a Pearson correlation coefficient of 0.928. Ablation experiments further confirmed the effectiveness of asynchronous alignment, reliability calibration, missing-modality compensation, economic modulation, and long-term graph memory. The proposed approach provides a temporally interpretable intelligent sensing method for monitoring computing center construction, evaluating industrial park expansion, allocating energy and communication infrastructure, and planning equipment supply.

## 1. Introduction

With the restructuring of globalization, the strengthening of technological sovereignty, and intensifying competition in artificial intelligence and semiconductor industries, the development of high-technology industries has evolved from an enterprise-level innovation issue into a complex systems problem jointly driven by policy, technology, capital, and infrastructure [1]. The establishment of AI computing centers, wafer fabrication facilities, and semiconductor industrial parks typically follows an evolutionary process from policy formulation and capital investment to technological accumulation and physical infrastructure deployment [2]. Understanding this process is essential for evaluating industrial policies, assessing industrial-chain relocation, identifying regional investment risks, and forecasting changes in the global technological landscape [3]. From a systems perspective, policy documents represent institutional intentions, patent and innovation networks capture technological accumulation, economic indicators such as FDI and trade reflect capital responses, while satellite imagery, nighttime light observations, and industrial thermal data provide direct evidence of physical implementation [4]. Integrating these heterogeneous information sources therefore provides a comprehensive basis for monitoring and forecasting high-technology industrial development [5]. Existing studies generally analyze these information sources independently. Policy research focuses on policy diffusion and institutional evolution, economic studies examine industrial growth through econometric models, innovation research relies on patent and collaboration networks, and remote sensing detects industrial expansion using land-use change and nighttime light observations [6,7]. Although these approaches are interpretable, they fail to model the interactions among institutional decisions, technological innovation, capital allocation, and physical construction. Consequently, policy documents cannot verify project implementation, remote sensing cannot explain underlying policy or financial drivers, and patent networks provide limited evidence for short-term investment dynamics [8]. Another challenge lies in the asynchronous and heterogeneous nature of industrial evolution [9]. Policy releases, capital inflows, patent growth, and infrastructure construction often occur at different stages, making fixed temporal windows insufficient for capturing policy-driven, capital-amplified, or technology-induced development patterns [10,11]. Meanwhile, multilingual policy documents, remote-sensing imagery, patent databases, and economic statistics exhibit varying levels of uncertainty due to linguistic bias, observation conditions, publication delays, and missing values [12]. Conventional feature concatenation and fixed-weight fusion are therefore unable to adapt to heterogeneous data quality and temporal inconsistency, resulting in semantic misalignment, redundant information, and noise propagation [13].

Recent advances in deep learning have established an effective foundation for jointly modeling heterogeneous industrial data from multiple sources [14]. Multilingual pretrained language models can extract policy themes, policy instruments, target industries, and implementation intensity from policy documents, while Vision Transformers identify building expansion, nighttime illumination, and thermal variations from multitemporal remote-sensing imagery. Graph neural networks characterize relationships among countries, institutions, patents, and technologies, whereas temporal models such as Transformers and TimesNet capture long-term trends and periodic dynamics in FDI, trade, and financial indicators [15]. Furthermore, cross-modal attention and contrastive learning enable unified representation learning across policy, technological, economic, and physical observations, while dynamic heterogeneous graph models facilitate the analysis of information propagation among countries, enterprises, and industrial parks [16]. Despite these advances, several limitations remain. Most existing studies assume temporal synchronization among different modalities, overlooking the heterogeneous response delays among policy, capital, patent activity, and physical construction [17]. Existing missing-modality and uncertainty-aware fusion methods generally focus on data availability or prediction uncertainty, while rarely considering data completeness, observation quality, temporal freshness, and cross-modal consistency in a unified manner [18]. Moreover, dynamic graph models are primarily developed for social networks and recommendation systems, with limited capability to model industrial relationships such as trade, FDI, supply-chain dependence, and patent collaboration. Their predictions are also seldom integrated with causal analysis, making it difficult to explain how policy interventions evolve into technological deployment and industrial capacity formation [19,20]. Recent studies have increasingly emphasized integrated intelligent systems that jointly model communication, sensing, computation, and knowledge. Representative frameworks have combined edge intelligence with multimodal sensing, integrated digital twins with generative artificial intelligence, and unified semantic communication with adaptive resource management to improve cross-modal consistency, system robustness, computational efficiency, and intelligent decision-making [21,22,23]. These developments demonstrate a clear trend toward system-level multimodal intelligence, yet comprehensive modeling of asynchronous industrial evolution, heterogeneous data quality, dynamic industrial relationships, and causal propagation remains largely unexplored.

To address these limitations, a multimodal semantic sensing and dynamic heterogeneous graph framework is proposed for monitoring the evolution of artificial intelligence computing infrastructure and the semiconductor industry. Policy documents are treated as social sensing streams, and institutional semantics are extracted using a multilingual language model. Satellite imagery, nighttime light observations, and industrial thermal information are used to characterize physical construction states. Technological innovation and knowledge diffusion are represented through a heterogeneous patent graph, whereas capital activities are modeled through FDI, trade, and financial time series. Within a unified embedding space, asynchronous semantic alignment is achieved through learnable soft temporal windows and cross-modal contrastive learning. Reliability-aware fusion is then performed according to data completeness, observation quality, temporal freshness, and predictive uncertainty. A dynamic heterogeneous graph consisting of countries, policy topics, technologies, enterprises, and industrial parks is subsequently constructed. Economically modulated temporal encoding and graph memory are introduced to characterize long-term dependencies within the declaration–investment–construction system. Finally, multitask prediction, Granger causality testing, and mediation-effect analysis are jointly employed to identify leading, lagging, and transmission relationships among institutions, capital, and technology. The main contributions are summarized as follows.

1. The lack of a unified theoretical perspective for understanding high-technology industrial evolution is addressed by establishing a closed-loop evolutionary paradigm that jointly models institutional intentions, capital allocation, technological innovation, and physical infrastructure, enabling policy diffusion and industrial development to be interpreted within a dynamic socio-technical system.
2. The temporal misalignment and heterogeneous reliability of multimodal industrial data are addressed through asynchronous semantic alignment and reliability-aware information fusion, allowing heterogeneous observations to remain robust under response delays, missing modalities, and varying data quality.
3. The limited capability of existing dynamic graph models to represent real-world industrial ecosystems is addressed by modeling trade, FDI, supply-chain dependence, and patent collaboration within a unified heterogeneous graph, enabling long-term cross-national interactions among policy, capital, and technology to be effectively characterized.
4. The limited interpretability of industrial evolution analysis is addressed by quantitatively distinguishing policy-led, capital-amplified, and technology-driven evolutionary pathways, providing explainable evidence for industrial policy evaluation, infrastructure monitoring, and development trend forecasting.

## 2. Related Work

### 2.1. Multimodal Social Sensing and Industrial-State Monitoring

Social sensing aims to continuously monitor social activities, economic operations, and industrial development by integrating heterogeneous data collected from governments, enterprises, public platforms, remote-sensing systems, and economic statistics [24]. Within this paradigm, policy documents and news reflect institutional intentions, remote-sensing imagery records physical construction and land-use changes, patent data capture technological innovation and knowledge diffusion [25], while FDI, trade, and financial indicators characterize capital flows and market dynamics [26]. These complementary information sources provide a comprehensive perspective for understanding high-technology industrial evolution [27]. Recent studies have also explored AI-driven sensing and data-centric monitoring for complex infrastructure systems. For example, Seo et al. [28] employed functional data analysis to characterize electricity-consumption patterns for intelligent monitoring of energy infrastructure systems. Although their application focuses on energy infrastructure, it similarly demonstrates that extracting informative patterns from heterogeneous temporal sensing data can improve infrastructure monitoring and intelligent system analysis. Recent studies have developed specialized sensing methods for different modalities. Multilingual pretrained language models extract policy semantics while reducing cross-language information loss [29]. Vision Transformers and multitemporal change-detection methods identify industrial expansion and infer regional economic activity from remote-sensing imagery [30]. Patent analysis relies on citation and collaboration networks to evaluate innovation capacity, whereas temporal economic models analyze investment dynamics using FDI, trade, and financial indicators [31,32]. These advances have substantially improved the perception capability of individual sensing modalities. Recent studies have also explored heterogeneous information integration for intelligent decision-making in complex industrial systems. For example, Jo et al. [33] proposed an AI-enabled optimization framework that jointly integrates multiple sources of operational information for green supplier selection and order allocation. Although their study focuses on supply-chain optimization rather than industrial-state monitoring, it further demonstrates that heterogeneous information fusion can enhance system-level reasoning and decision support in complex industrial environments. However, existing approaches remain largely modality-specific and therefore capture only isolated aspects of industrial evolution [34]. Policy documents reveal institutional intentions but cannot verify infrastructure deployment, whereas remote-sensing observations provide physical evidence without explaining the underlying policy or capital drivers [35,36]. Consequently, the unified modeling of institutional intentions, technological innovation, capital responses, and physical construction remains a fundamental challenge for intelligent monitoring of high-technology industries [37].

### 2.2. Cross-Modal Representation Learning and Reliability-Aware Fusion

The objective of cross-modal representation learning is to project heterogeneous data with different structures and semantics into a shared feature space [38], enabling complementary information from multiple modalities to be jointly exploited [39]. Early studies mainly relied on feature concatenation or late decision fusion [40], which either suffered from heterogeneous feature distributions or provided limited capability for modeling deep cross-modal interactions [41]. More recently, cross-attention, multimodal Transformers, and contrastive learning have significantly improved multimodal representation by enabling semantic interaction and alignment across policy documents, remote-sensing observations, patent activities, and economic indicators [42,43]. In particular, policy documents can serve as semantic anchors to associate subsequent investment growth, patent evolution, and infrastructure construction, facilitating the identification of how institutional intentions evolve into observable industrial activities [44,45]. Despite these advances, existing cross-modal methods generally assume temporal synchronization among different modalities, whereas policy implementation, capital inflows, patent growth, and physical construction typically occur with substantial response delays in high-technology industries [46]. Moreover, heterogeneous industrial data exhibit varying reliability due to cloud-contaminated remote-sensing imagery, delayed patent and economic statistics, and incomplete policy documents across regions [47]. Although missing-modality learning, uncertainty-aware fusion, and quality-aware weighting have alleviated some of these issues, they usually consider only missing data or prediction uncertainty while overlooking the joint effects of data completeness, observation quality, temporal freshness, and cross-modal consistency [48]. Therefore, robust industrial representation learning requires both asynchronous semantic alignment and reliability-aware adaptive fusion.

### 2.3. Dynamic Heterogeneous Graph Learning and Temporal Causal Analysis

Graph learning represents entities as nodes and their interactions as edges, enabling relational information to be learned through neighborhood propagation [49]. Graph convolutional and graph attention networks aggregate neighboring information [50], while heterogeneous graph neural networks further distinguish multiple node types and relation types [51]. Heterogeneous Graph Transformers extend this capability through type-specific attention, providing a unified framework for modeling complex industrial ecosystems [52]. To capture evolving industrial relationships, temporal graph models incorporate temporal encoding, dynamic graph updates, and historical memory to characterize long-term dependencies among policy implementation, capital allocation, and technological development [53,54]. In high-technology industries, however, information propagation is constrained by FDI flows, trade links, supply-chain dependence, and patent collaboration rather than random graph connectivity, requiring heterogeneous industrial graphs with dynamic edge weights and relation types [55]. For mechanism interpretation, Granger causality analysis identifies lead–lag relationships among policy, economic, and technological signals [56], while mediation analysis examines whether capital flows transmit the effects of policy interventions to technological deployment [57]. Despite these advances, existing dynamic graph models have mainly been developed for social networks, recommendation systems, and financial transactions with relatively regular temporal observations. By contrast, policy documents, remote-sensing imagery, patent records, and economic statistics exhibit heterogeneous sampling frequencies and irregular response delays [58]. Consequently, unified modeling of heterogeneous relations, dynamic edge evolution, nonlinear temporal effects, and economic mediation remains insufficiently explored, motivating the development of an economically modulated dynamic heterogeneous graph evolution framework [59].

## 3. Materials and Method

### 3.1. Data Collection

A multimodal dataset comprising policy documents, remote-sensing imagery, patent relations, and economic time series was constructed to characterize the evolution of artificial intelligence computing infrastructure and the semiconductor industry. Data were collected from January 2018 to December 2025, covering countries and regions with intensive artificial intelligence and semiconductor activities, including China, the United States, the European Union, Japan, South Korea, Singapore, and India, as shown in Table 1. Policy documents were primarily collected from national government portals, industrial and information technology authorities, science and technology agencies, development and planning departments, and publicly accessible local government platforms. The collected documents included national strategies, industrial development plans, investment subsidies, tax incentives, technology-control measures, export restrictions, industrial-park construction plans, and approvals for major projects. A self-developed multilingual policy text sensor was employed to perform incremental webpage crawling, attachment parsing, and publication-date verification. Incremental updates were conducted daily, while document titles, full text, issuing authorities, publication dates, applicable regions, policy instruments, and target industries were retained. A total of 46,280 policy documents were collected. Duplicate reposts, pages without valid textual content, and documents with highly similar content were removed through a combination of document fingerprinting and semantic-similarity-based deduplication. The official publication date of each document was subsequently used as the temporal anchor for cross-modal matching.

Remote-sensing data were mainly obtained from Copernicus Sentinel-2, USGS Landsat 8/9, and nighttime light products acquired by Suomi NPP and NOAA-20. Sentinel-2 MSI provides visible, near-infrared, and shortwave-infrared bands at spatial resolutions of 10–20 m and was primarily used to identify industrial-park expansion, land-surface hardening, and changes in large-scale buildings. Landsat 8/9 OLI/TIRS provides multispectral imagery and land-surface temperature observations at a spatial resolution of 30 m and was used to monitor industrial thermal anomalies and long-term land-use changes. VIIRS DNB nighttime light observations were used to characterize industrial-park operating intensity and regional infrastructure activity. Images were downloaded according to predefined buffer zones surrounding major industrial parks, wafer fabrication facilities, and artificial intelligence computing centers. The revisit interval ranged from 5 to 16 d, and 18,640 valid scenes were retained. Cloud and shadow detection, radiometric correction, geometric registration, spatial cropping, and monthly compositing were performed after acquisition. Metadata, including sensor type, acquisition time, cloud coverage, spatial resolution, and geographic coordinates, were also preserved.

Patent data were primarily collected from publicly accessible patent retrieval systems, including WIPO PATENTSCOPE, EPO Open Patent Services, the China National Intellectual Property Administration, USPTO, J-PlatPat, and KIPRIS. The collection scope covered technological fields such as artificial intelligence chips, advanced semiconductor processes, packaging and testing, lithography equipment, memory devices, data centers, and high-performance computing. Joint searches based on IPC/CPC classification codes, technical keywords, and applicants were conducted to retrieve patent application numbers, publication dates, applicants, inventors, countries of origin, technological classifications, citation relations, patent-family relations, and co-application relations. A total of 1,342,760 patent records were obtained. After patent-title disambiguation, institution-name normalization, and patent-family consolidation, a heterogeneous relation graph involving countries, enterprises, patents, and technological topics was constructed. Economic time-series data were collected from UN Comtrade, UNCTAD, the World Bank, the International Monetary Fund, the OECD, national statistical agencies, and major stock exchanges. The collected indicators included foreign direct investment inflows and outflows, bilateral trade volumes, imports and exports of high-technology products, fixed-asset investment, corporate capital expenditure, industrial indices, stock prices, market volatility, interest rates, and exchange rates. All indicators were harmonized at monthly or quarterly frequencies, resulting in 312,480 valid observations. Ground-truth construction events were established through a multi-source verification procedure. Candidate events were first collected from official government announcements, industrial park approval documents, enterprise investment disclosures, and publicly available project reports. These candidates were subsequently verified using multi-temporal remote-sensing observations, including visible imagery, nighttime-light data, and land-surface thermal measurements. A construction event was labeled as positive only when documentary evidence and remote-sensing observations consistently indicated the commencement of physical site development. Continuous construction-intensity labels were generated using a normalized composite index integrating newly developed construction area, nighttime-light variation, land-surface thermal variation, and investment-scale indicators. All candidate labels were independently reviewed by two annotators with expertise in remote sensing and industrial policy analysis, and disagreements were resolved through consensus with a senior reviewer. Negative samples were defined as regions exhibiting neither officially documented construction activities nor significant remote-sensing changes throughout the observation period. Samples with conflicting evidence across different information sources or inconclusive remote-sensing observations were categorized as ambiguous cases and excluded from both model training and evaluation. This strategy reduced label noise and improved the consistency and reliability of the supervised learning dataset.

### 3.2. Data Augmentation

The fundamental principle of data augmentation is to generate additional training samples through constrained transformations without altering the core semantics or ground-truth labels of the original samples. This process expands the coverage of the data distribution and reduces model dependence on specific linguistic expressions, observation conditions, and levels of data completeness. For multimodal industrial data composed of policy documents, remote-sensing imagery, patent graphs, and economic time series, modality-specific structures and invariance constraints differ substantially. Consequently, a uniform augmentation strategy cannot be applied across all modalities. Let the original sample of modality *m* be denoted by $x^{m}$, and let the corresponding augmentation operator be denoted by $T_{m}$. The augmented sample is expressed as

(1) $${\tilde{x}}^{m}=T_{m}\left(x^{m};\theta_{m}\right),$$

where *m* denotes the modality type and $\theta_{m}$ represents the augmentation intensity or transformation parameters. The augmentation procedure is constrained to preserve critical information related to industrial topics, temporal attributes, and entity relationships between ${\tilde{x}}^{m}$ and $x^{m}$. Policy-text augmentation is designed to improve model adaptability to linguistic variation, policy-expression diversity, and multilingual content. Semantically equivalent samples are generated through synonym substitution, multilingual back-translation, local paragraph-order perturbation, noncritical sentence masking, and entity-preserving text rewriting. Policy documents describing the same industrial topic in different languages can also be treated as cross-lingual positive pairs to enhance linguistic consistency within the shared semantic space. Let the encoded representation of a policy document be denoted by $h_{t}$ and the representation of its augmented counterpart by ${\tilde{h}}_{t}$. Their semantic consistency constraint is defined as

(2) $$L_{\mathrm{text}}=1-\frac{h_{t}^{T}{\tilde{h}}_{t}}{{∥h_{t}∥}_{2}{∥{\tilde{h}}_{t}∥}_{2}}.$$

This loss limits semantic deviation between the original and augmented text representations. During augmentation, critical entities, including countries, institutions, industrial targets, policy orientations, monetary values, dates, and implementation periods, are strictly preserved to prevent synonym replacement or back-translation from altering factual policy information. Remote-sensing image augmentation simulates variations in imaging angle, illumination conditions, sensor noise, and cloud obstruction to improve the robustness of the visual encoder under complex observation environments. Random cropping, small-angle rotation, brightness and contrast perturbation, Gaussian noise injection, channel masking, simulated cloud occlusion, and random temporal-frame dropping are applied to the image sequences. The general transformation is formulated as

(3) $${\tilde{I}}_{t}=M_{t}⊙\left(\alpha G\left(I_{t}\right)+\beta +\epsilon \right),$$

where $I_{t}$ denotes the remote-sensing image acquired at time *t*, G(·) represents geometric transformations such as cropping, flipping, or rotation, and α and β control image contrast and brightness, respectively. The noise term satisfies $\epsilon ∼N(0,\sigma^{2})$, while $M_{t}$ denotes a pixel-level, channel-level, or temporal-frame mask. For regions in which road orientation, coastlines, and industrial-park layouts carry explicit geographical meaning, vertical flipping and large-angle rotation are restricted to prevent distortion of the actual spatial structure. Patent-graph augmentation perturbs local graph structures and node attributes while preserving the principal knowledge-diffusion pathways and relation types. This strategy reduces excessive model dependence on a limited number of nodes or edges. Low-weight edge dropping, node-attribute masking, subgraph sampling, temporal-edge perturbation, topic-consistent contrastive sampling, and relation-preserving edge reconstruction are adopted. Let the original adjacency matrix associated with relation type *r* be denoted by $A^{r}$, where *r* may represent citation, collaboration, or joint application. The augmented adjacency matrix is defined as

(4) $${\tilde{A}}^{r}=A^{r}⊙B^{r}+R^{r},$$

where $R^{r}$ represents reconstructed edges generated under constraints on relation type and technological-topic consistency. Highly cited edges, backbone relationships among core patents, and cross-national collaboration chains are assigned relatively low removal probabilities to ensure that the augmented graph remains consistent with realistic technological diffusion patterns. Economic time-series augmentation is employed to improve model adaptability to statistical noise, short-term fluctuations, and local data incompleteness. Random temporal masking, sliding-window sampling, small-amplitude Gaussian perturbation, temporal-segment cropping, frequency-domain perturbation, and interpolation between adjacent observations are applied. For an original economic sequence $x_{t}$, the augmented sequence is expressed as

(5) $${\tilde{x}}_{t}=m_{t}\left(x_{t}+\epsilon_{t}\right)+\left(1-m_{t}\right){\hat{x}}_{t},$$

where $m_{t}\in 0,1$ denotes the temporal mask, $\epsilon_{t}∼N\left(0,\sigma_{x}^{2}\right)$ represents a constrained random perturbation, and ${\hat{x}}_{t}$ denotes the interpolated value derived from adjacent observations. Missing-modality augmentation randomly masks one or more data sources during training to simulate practical conditions such as incomplete policy records, cloud-obstructed remote-sensing imagery, delayed patent disclosure, and missing economic statistics. Let $z_{m}$ denote the availability mask of modality *m*. The augmented input is formulated as

(6) $${\tilde{x}}^{m}=z_{m}x^{m}+\left(1-z_{m}\right)e_{\mathrm{miss}}^{m},\;z_{m}∼\mathrm{Bernoulli}\left(1-p_{m}\right),$$

where $p_{m}$ denotes the missing probability of modality *m*, and $e_{\mathrm{miss}}^{m}$ represents a learnable missing-modality token. Correlated modalities can also be jointly masked during training to simulate extreme conditions in which multiple data sources are simultaneously unavailable. Through this strategy, predictions can be generated from the remaining modalities and historical states, thereby improving robustness under uneven global industrial data quality and incomplete multimodal observations.

### 3.3. Proposed Method

#### 3.3.1. Overall Architecture

The preprocessed policy documents, remote-sensing imagery, patent relation graphs, and economic time series are first passed through a multilingual policy encoder, a visual spatiotemporal encoder, a patent graph encoder, and an economic temporal encoder, respectively. Modality-specific representations of institutional intentions, physical construction, technological diffusion, and capital activities are thereby extracted. The representations generated by the four encoders are subsequently mapped through independent projection layers into a shared semantic space with a unified dimensionality and are then forwarded to the asynchronous cross-modal semantic alignment module. Policy announcements or major industrial events are treated as temporal anchors, and the most relevant economic, patent, and remote-sensing observations are retrieved from candidate windows of different lengths. Soft temporal matching is then applied to generate aligned cross-modal representations and estimate their corresponding response delays. The aligned representations are subsequently processed by the reliability-aware fusion module. Data completeness, observation quality, temporal freshness, model uncertainty, and cross-modal consistency are jointly considered to generate sample-specific modality weights. Missing modalities are supplemented through uncertainty-constrained auxiliary reconstruction, resulting in an integrated state representation at the national or regional level. The fused representations are then written into a dynamic heterogeneous graph composed of countries, policy topics, technological entities, institutions, patents, and industrial parks. The economic modulation component adjusts the temporal decay and propagation strength of different relations according to investment activity, market pressure, and capital flows. Relation-aware graph attention aggregates neighboring information along trade, FDI, patent collaboration, and supply-chain connections. A long-term graph memory mechanism further integrates current observations with historical states to produce the final dynamic node representations. These representations are finally passed to multiple task-specific prediction heads to jointly predict policy diffusion, investment changes, patent activities, infrastructure construction intensity, and the formation of cross-national industrial relationships. The symbols used in the text are summarized in Table 2.

#### 3.3.2. Asynchronous Cross-Modal Semantic Alignment Module (ACAM)

The asynchronous cross-modal semantic alignment module receives the policy, remote-sensing, patent graph, and economic features generated by the corresponding modality encoders.

**Figure 1:**
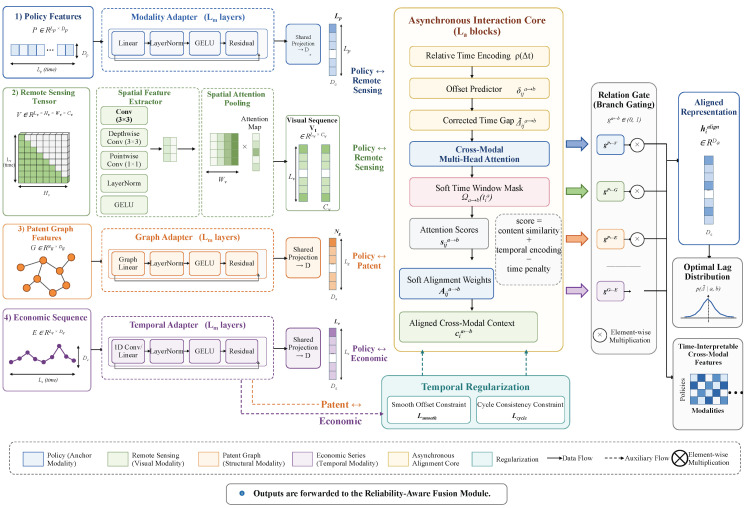
The asynchronous cross-modal semantic alignment module aligns policy text, remote-sensing imagery, patent graphs, and economic sequences through modality adaptation, learnable temporal offsets, cross-modal attention, soft temporal windows, and temporal regularization to produce time-interpretable representations.

As shown in Figure 1, the policy feature sequence, remote-sensing spatiotemporal features, patent graph features, and economic temporal features are represented as follows:

(7) $$P\in R^{L_{p}\times D_{p}},\;V\in R^{L_{v}\times H_{v}\times W_{v}\times C_{v}},\;G\in R^{N_{g}\times D_{g}},\;E\in R^{L_{e}\times D_{e}}.$$

Here, $L_{p}$, $L_{v}$, and $L_{e}$ denote the lengths of the policy, visual, and economic sequences, respectively. $H_{v}$, $W_{v}$, and $C_{v}$ denote the height, width, and channel dimension of the remote-sensing feature map, respectively, while $N_{g}$ denotes the number of nodes in the patent graph. The spatial dimensions of the remote-sensing features are first compressed through spatial attention pooling. The positional attention weight is defined as

(8) $$\alpha_{t,h,w}^{v}=\frac{\exp\left(q_{v}^{T}V_{t,h,w}\right)}{\sum_{h^{\prime }=1}^{H_{v}}\sum_{w^{\prime }=1}^{W_{v}}\exp\left(q_{v}^{T}V_{t,h^{\prime },w^{\prime }}\right)}.$$

The visual representation associated with the *t*-th temporal window is calculated as

(9) $$v_{t}=\sum_{h=1}^{H_{v}}\sum_{w=1}^{W_{v}}\alpha_{t,h,w}^{v}V_{t,h,w}.$$

Through this operation, a remote-sensing feature map of size $H_{v}\times W_{v}\times C_{v}$ is transformed into a temporal token with a channel width of $C_{v}$, while spatial information associated with building expansion, nighttime illumination changes, and thermal anomalies is retained. Policy, visual, patent, and economic features are then processed by modality-specific adaptation networks consisting of $L_{m}$ layers. Each layer contains a linear transformation, layer normalization, nonlinear activation, dropout, and a residual connection. The hidden width is denoted by $D_{h}$, and the output width is unified as $D_{a}$. The computation performed at the *l*-th layer is expressed as

(10) $$Z_{m}^{\left(l\right)}=Z_{m}^{\left(l-1\right)}+W_{m,2}^{\left(l\right)}\mathrm{GELU}\left(W_{m,1}^{\left(l\right)}\mathrm{LN}\left(Z_{m}^{\left(l-1\right)}\right)+b_{m,1}^{\left(l\right)}\right)+b_{m,2}^{\left(l\right)}.$$

The adapted features are subsequently projected into a unified semantic space through modality-specific linear mappings:

(11) $${\hat{Z}}_{m}=\mathrm{LN}\left(Z_{m}^{\left(L_{m}\right)}W_{m}^{\mathrm{proj}}+b_{m}^{\mathrm{proj}}+e_{m}\right).$$

Here, $e_{m}$ denotes the modality-type embedding, which preserves structural distinctions among different signal sources within the shared semantic space. The alignment network is composed of $L_{a}$ asynchronous interaction blocks. Each block consists of multi-head cross-modal attention, a temporal-offset predictor, a feed-forward network, residual connections, and normalization layers. For the *i*-th temporal token in anchor modality *a* and the *j*-th temporal token in target modality *b*, a content-dependent temporal offset is first estimated as

(12) $$\delta_{ij}^{a\to b}=w_{\delta ,ab}^{T}\tanh\left(W_{\delta ,a}{\hat{z}}_{a,i}+W_{\delta ,b}{\hat{z}}_{b,j}+W_{\delta ,t}\rho \left(t_{j}^{b}-t_{i}^{a}\right)+b_{\delta ,ab}\right).$$

The function ρ(·) denotes the relative temporal encoding function. The predicted offset is used to correct the original temporal distance between the two modalities:

(13) $${\tilde{\Delta }}_{ij}^{a\to b}=t_{j}^{b}-t_{i}^{a}-\delta_{ij}^{a\to b}.$$

The attention score jointly determined by content similarity, modality relations, and temporal distance is calculated as

(14) $$s_{ij,r}^{a\to b}=\frac{{\left(q_{i,r}^{a}\right)}^{T}k_{j,r}^{b}}{\sqrt{D_{r}}}+{\left(u_{r}^{a\to b}\right)}^{T}\rho \left({\tilde{\Delta }}_{ij}^{a\to b}\right)-\lambda_{r}^{a\to b}\left|{\tilde{\Delta }}_{ij}^{a\to b}\right|.$$

Here, $D_{r}$ denotes the feature width of an individual attention head, and $\lambda_{r}^{a\to b}$ is a learnable temporal-decay coefficient. The normalized asynchronous attention weight is then obtained as

(15) $$A_{ij,r}^{a\to b}=\frac{\exp\left({\bar{s}}_{ij,r}^{a\to b}/\tau_{ab}\right)}{\sum_{k=1}^{L_{b}}\exp\left({\bar{s}}_{ik,r}^{a\to b}/\tau_{ab}\right)}.$$

The policy state is treated as the primary semantic anchor. Policy–remote-sensing, policy–patent, policy–economic, and patent–economic interaction branches are constructed separately. Independent parameters are assigned to the different branches so that the short-term influence of policy on capital activity, its medium-term influence on patent and technological activity, and its long-term influence on physical infrastructure construction can be learned separately. The gating weight associated with each interaction branch is calculated as

(16) $$g_{i}^{a\leftarrow b}=\frac{\exp\left(w_{g,ab}^{T}\tanh\left(W_{g,a}{\hat{z}}_{a,i}+W_{g,b}c_{i}^{a\leftarrow b}+b_{g,ab}\right)\right)}{\sum_{n\in M\setminus a}\exp\left(w_{g,an}^{T}\tanh\left(W_{g,a}{\hat{z}}_{a,i}+W_{g,n}c_{i}^{a\leftarrow n}+b_{g,an}\right)\right)}.$$

The outputs of the multiple interaction branches are integrated through a residual structure:

(17) $$h_{i}^{\mathrm{align}}=\mathrm{LN}\left(W_{r}^{a}{\hat{z}}_{a,i}+\sum_{b\in M\setminus a}g_{i}^{a\leftarrow b}c_{i}^{a\leftarrow b}\right).$$

A temporal-offset smoothness constraint is introduced to prevent irregular variations in estimated delays between adjacent temporal windows:

(18) $$L_{\mathrm{smooth}}=\sum_{\left(a,b\right)\in R_{a}}\sum_{i=2}^{L_{a}}\left|{\bar{\delta }}_{i}^{a\to b}-{\bar{\delta }}_{i-1}^{a\to b}\right|.$$

A cyclic offset constraint is further introduced to maintain consistency between forward and reverse temporal relationships:

(19) $$L_{\mathrm{cycle}}=\sum_{\left(a,b\right)\in R_{a}}\sum_{i=1}^{L_{a}}\left|{\bar{\delta }}_{i}^{a\to b}+{\bar{\delta }}_{\pi (i)}^{b\to a}\right|.$$

Here, π(i) denotes the corresponding position in the target modality determined by the forward attention distribution. The temporal regularization loss is defined as

(20) $$L_{\mathrm{temporal}}=\eta_{1}L_{\mathrm{smooth}}+\eta_{2}L_{\mathrm{cycle}}.$$

The proposed soft temporal alignment includes conventional hard alignment as a special case.

#### 3.3.3. Reliability-Aware Multimodal Adaptive Fusion Module (RAMF)

The reliability-aware multimodal adaptive fusion module receives the policy, remote-sensing, patent, and economic representations generated by the asynchronous cross-modal semantic alignment module. The contribution of each modality is dynamically determined according to its actual observation quality in each sample.

**Figure 2:**
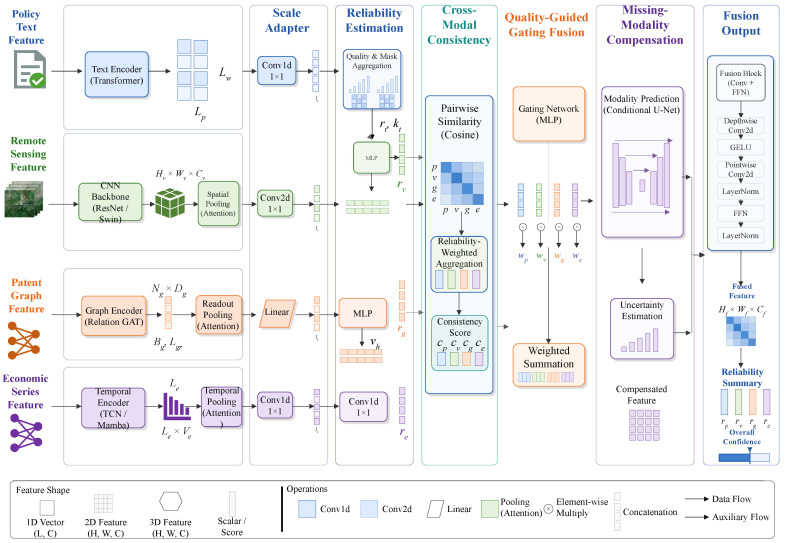
The reliability-aware multimodal adaptive fusion module adaptively integrates policy, remote-sensing, patent, and economic features through scale adaptation, modality-quality estimation, cross-modal consistency calibration, quality-guided gating, and missing-modality compensation, while producing fused representations and overall confidence estimates.

As shown in Figure 2, let the modality set be denoted by M, and let the aligned feature of modality *m* be represented by $X^{m}\in R^{H_{m}\times W_{m}\times C_{m}}$, where $H_{m}$ and $W_{m}$ denote the spatial or temporal structural dimensions and $C_{m}$ denotes the channel width. For sequential modalities such as policy, patent, and economic data, $H_{m}$ can be interpreted as the temporal-window length and $W_{m}$ as the number of local semantic units. For remote-sensing data, $H_{m}$ and $W_{m}$ correspond to the height and width of the feature map, respectively. Each modality is first processed by an independent scale-adaptation network and transformed into a unified size of $H_{f}\times W_{f}\times C_{f}$:

(21) $$F^{m}=P_{m}\left(X^{m};\Theta_{m}^{p}\right),\;F^{m}\in R^{H_{f}\times W_{f}\times C_{f}}.$$

The operator $P_{m}\left(\cdot \right)$ consists of $L_{p}$ adaptation blocks. Each block contains channel mapping, depthwise separable convolution, normalization, nonlinear activation, and a residual connection. The input and output channel dimensions of the *l*-th layer are denoted by $C_{l-1}^{m}$ and $C_{l}^{m}$, respectively, while the convolutional kernel size is $K_{l}^{m}\times K_{l}^{m}$. The final channel dimension satisfies $C_{L_{p}}^{m}=C_{f}$. For modalities without a two-dimensional spatial structure, the two-dimensional convolution is replaced by a one-dimensional convolution along the temporal dimension. The scale-adapted features are subsequently passed to the reliability-estimation branch. This branch consists of an $L_{r}$-layer quality-encoding network and a reliability output layer. Its inputs include modality features, missing-data masks, observation quality, temporal freshness, and predictive uncertainty. The quality descriptor of modality *m* is defined as

(22) $$q^{m}={\left[c^{m},o^{m},f^{m},u^{m},s^{m}\right]}^{T},$$

where $c^{m}$ denotes data completeness, $o^{m}$ denotes observation quality, $f^{m}$ denotes temporal freshness, $u^{m}$ denotes model uncertainty, and $s^{m}$ denotes alignment stability generated by the asynchronous alignment module. For the policy modality, observation quality is determined by document completeness, policy certainty, and entity-recognition confidence. For the remote-sensing modality, it is determined by the effective-pixel ratio, cloud coverage, and registration error. For the patent modality, network density, institution-disambiguation confidence, and publication delay are considered. For the economic modality, the missing-value ratio, statistical revision magnitude, and publication delay are incorporated. The modality reliability score is generated through a bounded mapping:

(23) $$\rho^{m}=\frac{1}{1+\exp\left(-w_{\rho ,m}^{T}r_{L_{r}}^{m}-b_{\rho ,m}\right)}.$$

To avoid estimating reliability exclusively from modality-specific quality indicators, a cross-modal consistency-correction branch is further introduced. The weighted distance between any two modalities in the shared feature space is calculated as

(24) $$d^{m,n}={\left(g^{m}-g^{n}\right)}^{T}\sum_{m,n}^{-1}\left(g^{m}-g^{n}\right),$$

where $\Sigma_{m,n}$ denotes a learnable covariance matrix associated with the modality pair. The consistency coefficient between modality *m* and the remaining modalities is defined as

(25) $$\chi^{m}=\frac{\sum_{\begin{array}{c} n\in M\\ n\neq m \end{array}}\rho^{n}\exp\left(-d^{m,n}\right)}{\sum_{\begin{array}{c} n\in M\\ n\neq m \end{array}}\rho^{n}+\varepsilon }.$$

When a modality exhibits a state similar to those of several highly reliable modalities, $\chi^{m}$ increases. In contrast, substantial disagreement with other credible observations results in a lower consistency coefficient. The reliability score and consistency coefficient are jointly processed by a gating network to generate sample-specific fusion weights:

(26) $$\zeta^{m}=a_{m}^{T}\mathrm{Act}\left(B_{m}g^{m}+C_{m}q^{m}+D_{m}\left[\rho^{m};\chi^{m}\right]+b_{m}\right).$$

(27) $$\omega^{m}=\frac{\left(\rho^{m}\chi^{m}+\varepsilon \right)\exp\left(\zeta^{m}\right)}{\sum_{n\in M}\left(\rho^{n}\chi^{n}+\varepsilon \right)\exp\left(\zeta^{n}\right)}.$$

The resulting weights account simultaneously for modality content, observation quality, and cross-modal consistency. For example, when remote-sensing imagery is severely affected by cloud coverage, the reduced effective-pixel ratio decreases $\rho^{v}$. Similarly, policy documents that are complete but lack explicit implementation commitments receive limited weights because of their low policy certainty. In contrast to fixed-weight fusion, the proposed mechanism produces different modality-weight distributions for different countries, temporal windows, and industrial events. When modality *m* is missing, its latent representation is conditionally reconstructed from the remaining available modalities and the historical fused state:

(28) $${\hat{F}}^{m}=D_{m}\left(\mathrm{Concat}\left[{\left\{\omega^{n}F^{n}\right\}}_{n\neq m},H_{t-1}\right]\right).$$

The reconstruction network $D_{m}\left(\cdot \right)$ consists of an $L_{d}$-layer encoder and an $L_{d}$-layer decoder. The channel widths of the encoding layers are denoted by $C_{d,1},C_{d,2},\ldots ,C_{d,L_{d}}$, while a symmetric decoding structure restores the feature map to $H_{f}\times W_{f}\times C_{f}$. Reconstructed representations are therefore prevented from being treated as equivalent to directly observed evidence. The final fused feature is obtained through channel concatenation, pointwise transformation, and residual aggregation:

(29) $$H_{t}=F\left(\mathrm{Concat}\left[\omega^{1}{\bar{F}}^{1},\omega^{2}{\bar{F}}^{2},\ldots ,\omega^{\left|M\right|}{\bar{F}}^{\left|M\right|}\right]\right).$$

The fusion operator F(·) is composed of $L_{f}$ channel-fusion blocks. Its input channel dimension is $\left|M\right|C_{f}$, the hidden-channel width is $C_{h}^{f}$, and the output-channel dimension is $C_{o}^{f}$. The proposed reliability estimation can be interpreted as a data-driven approximation of inverse-variance weighting.

#### 3.3.4. Economically Modulated Dynamic Heterogeneous Graph Evolution Module (EM-HTG)

The economically modulated dynamic heterogeneous graph evolution module receives the national- or regional-level integrated state representations generated by the reliability-aware fusion module. Policy topics, technological topics, enterprises and institutions, patents, industrial parks, and countries are organized into a time-varying heterogeneous graph.

**Figure 3:**
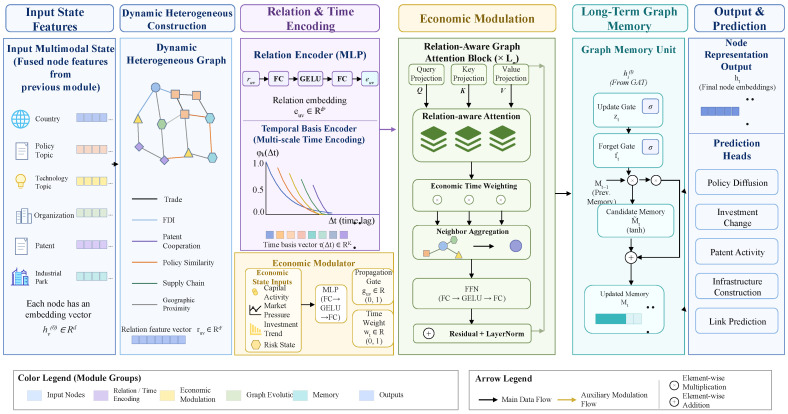
The economically modulated dynamic heterogeneous graph evolution module integrates relation and temporal encoding, economic-state gating, relation-aware graph attention, and long-term graph memory to model time-varying interactions among countries, policies, technologies, organizations, patents, and industrial parks for multitask prediction.

As shown in Figure 3, let the graph associated with temporal window *t* be denoted by $G_{t}$, the node-type set by Q, and the relation-type set by R. The feature matrix of nodes belonging to type *q* is represented as $X_{t}^{q}\in R^{N_{q}\times C_{q}}$, where $N_{q}$ denotes the number of nodes and $C_{q}$ denotes the input-channel dimension. Relation *r* connects source-node type s(r) and destination-node type d(r), and its adjacency matrix is represented by $A_{t}^{r}\in R^{N_{s(r)}\times N_{d(r)}}$. The height and width of this matrix correspond to the numbers of source and destination nodes, respectively. The module consists of $L_{g}$ relation-aware graph evolution blocks, an economic-state modulator, and a long-term graph memory unit. The node-channel width at the *l*-th layer is denoted by $C_{l}$, the number of multi-head relation-attention heads is denoted by $K_{l}$, and the width of an individual head is denoted by $D_{l}$. The economic-state modulator receives the capital activity, market pressure, investment trend, and risk state associated with the source and destination nodes and generates a relation-propagation gate. Let the economic representations of the two endpoint nodes be denoted by $x_{u,t}^{e}$ and $x_{v,t}^{e}$. The economic modulation coefficient is defined as

(30) $$\gamma_{uv,t}^{r}=\mathrm{sigmoid}\left({\left(w_{r}^{\gamma }\right)}^{T}\mathrm{GELU}\left(W_{r,u}^{\gamma }x_{u,t}^{e}+W_{r,v}^{\gamma }x_{v,t}^{e}+W_{r,z}^{\gamma }z_{uv,t}^{r,L_{e}}+b_{r}^{\gamma }\right)\right).$$

To capture both short-term capital responses and long-term industrial accumulation, the temporal interval $\Delta t_{uv}$ is mapped to a set of multiscale temporal basis functions:

(31) $$\phi_{uv,t}^{r}=\left[\exp\left(-\xi_{r,1}\Delta t_{uv}\right),\exp\left(-\xi_{r,2}\Delta t_{uv}\right),\ldots ,\exp\left(-\xi_{r,B}\Delta t_{uv}\right)\right].$$

The economic state further controls the mixture proportions assigned to different temporal scales:

(32) $$\beta_{uv,t}^{r}=\mathrm{softmax}\left(W_{r}^{\beta }\left[x_{u,t}^{e};x_{v,t}^{e};z_{uv,t}^{r,L_{e}}\right]+b_{r}^{\beta }\right).$$

The final economically modulated temporal weight is expressed as

(33) $$\vartheta_{uv,t}^{r}=\gamma_{uv,t}^{r}\sum_{b=1}^{B}\beta_{uv,t,b}^{r}\phi_{uv,t,b}^{r}.$$

When FDI inflows are active, industrial investment is stable, and market pressure is low, larger proportions can be assigned to medium- and long-term propagation components. When capital contracts or market risk increases, the influence of outdated relations is attenuated by the modulation mechanism. At the *l*-th layer, the *k*-th attention head independently projects the destination-node feature, source-node feature, and relation feature into query, key, and value spaces:

(34) $$q_{v,t}^{r,l,k}=H_{v,t}^{d(r),l-1}W_{Q}^{r,l,k}.$$

(35) $$k_{u,t}^{r,l,k}=H_{u,t}^{s(r),l-1}W_{K}^{r,l,k}+z_{uv,t}^{r,L_{e}}W_{K,e}^{r,l,k}.$$

(36) $$v_{u,t}^{r,l,k}=H_{u,t}^{s(r),l-1}W_{V}^{r,l,k}+z_{uv,t}^{r,L_{e}}W_{V,e}^{r,l,k}.$$

The relation-attention score is jointly determined by node content, relation features, and the economically modulated temporal weight:

(37) $$a_{uv,t}^{r,l,k}=\frac{q_{v,t}^{r,l,k}{\left(k_{u,t}^{r,l,k}\right)}^{T}}{\sqrt{D_{l}}}+\eta_{r,l,k}\log\left(\vartheta_{uv,t}^{r}+\epsilon \right).$$

The attention score is normalized over all heterogeneous neighbors of the destination node:

(38) $$\alpha_{uv,t}^{r,l,k}=\frac{\exp\left(a_{uv,t}^{r,l,k}\right)}{\sum_{r^{\prime }\in R}\sum_{u^{\prime }\in N_{r^{\prime }}\left(v\right)}\exp\left(a_{u^{\prime }v,t}^{r^{\prime },l,k}\right)}.$$

The relational message aggregated by the *k*-th attention head is

(39) $$m_{v,t}^{l,k}=\sum_{r\in R}\sum_{u\in N_{r}\left(v\right)}\alpha_{uv,t}^{r,l,k}\vartheta_{uv,t}^{r}v_{u,t}^{r,l,k}.$$

The multi-head messages are concatenated and passed through an output projection, a residual connection, and a feed-forward network to obtain the current graph state:

(40) $${\tilde{H}}_{v,t}^{l}=\mathrm{LayerNorm}\left(H_{v,t}^{l-1}+\mathrm{Concat}\left[m_{v,t}^{l,1},\ldots ,m_{v,t}^{l,K_{l}}\right]W_{O}^{l}\right).$$

(41) $$H_{v,t}^{l}=\mathrm{LayerNorm}\left({\tilde{H}}_{v,t}^{l}+{\mathrm{FFN}}_{l}\left({\tilde{H}}_{v,t}^{l}\right)\right).$$

The long-term graph memory unit has a channel width of $C_{M}$ and retains accumulated policy, capital, technological, and construction states for each node. The current graph representation and historical memory are jointly used to generate an input gate, a retention gate, and a candidate memory:

(42) $$i_{v,t}=\mathrm{sigmoid}\left(H_{v,t}^{L_{g}}W_{i}+M_{v,t-1}U_{i}+b_{i}\right).$$

(43) $$f_{v,t}=\mathrm{sigmoid}\left(H_{v,t}^{L_{g}}W_{f}+M_{v,t-1}U_{f}+b_{f}\right).$$

(44) $${\tilde{M}}_{v,t}=\tanh\left(H_{v,t}^{L_{g}}W_{M}+M_{v,t-1}U_{M}+b_{M}\right).$$

(45) $$M_{v,t}=f_{v,t}⊙M_{v,t-1}+i_{v,t}⊙{\tilde{M}}_{v,t}.$$

The final node output is generated by jointly incorporating the current graph state, long-term memory, and economic state:

(46) $$Y_{v,t}=\left[H_{v,t}^{L_{g}};M_{v,t};x_{v,t}^{e}\right]W_{y}+b_{y}.$$

The proposed module is permutation equivariant with respect to relation propagation. Let $\Pi_{q}$ denote an arbitrary permutation matrix within node type *q*. The permuted node-feature and relation matrices are defined as

(47) $$X_{t}^{q\prime }=\Pi_{q}X_{t}^{q}.$$

(48) $$A_{t}^{r\prime }=\Pi_{s(r)}A_{t}^{r}\Pi_{d(r)}^{T}.$$

Because attention normalization is performed only over the neighborhood of each destination node and neighboring messages are aggregated through summation, a permutation of node order does not alter the underlying message set. The propagation operation at layer *l* therefore satisfies

(49) $$H_{t}^{q,l\prime }=\Pi_{q}H_{t}^{q,l}.$$

By mathematical induction over the graph layers, the final output satisfies

(50) $$Y_{t}^{q\prime }=\Pi_{q}Y_{t}^{q}.$$

This property indicates that model predictions are independent of the order in which countries, enterprises, patents, or industrial-park nodes are arranged in the input matrices. Furthermore, the economic modulation coefficient, temporal basis functions, and attention weights are nonnegative and bounded:

(51) $$0\leq \gamma_{uv,t}^{r}\leq 1.$$

(52) $$0<\phi_{uv,t,b}^{r}\leq 1.$$

(53) $$\sum_{r\in R}\sum_{u\in N_{r}\left(v\right)}\alpha_{uv,t}^{r,l,k}=1.$$

The norm of the single-head aggregated message therefore satisfies

(54) $${\left|m_{v,t}^{l,k}\right|}_{2}\leq \max_{r,u}{\left|v_{u,t}^{r,l,k}\right|}_{2}.$$

Since the attention coefficients are normalized by the softmax operation, $\sum_{u}\alpha_{uv,t}^{r}=1$, and the economic modulation factor is generated through a sigmoid gate, $0\leq \theta_{uv,t}^{r}\leq 1$. Therefore, the aggregation coefficients satisfy $0\leq \alpha_{uv,t}^{r}\theta_{uv,t}^{r}\leq \alpha_{uv,t}^{r}$. Applying the triangle inequality and the convexity of weighted sums yields

(55) $${\left|m_{v,t}^{l,k}\right|}_{2}\leq \sum_{r,u}\alpha_{uv,t}^{r}\theta_{uv,t}^{r}{\left|v_{u,t}^{r,l,k}\right|}_{2}\leq \max_{r,u}{\left|v_{u,t}^{r,l,k}\right|}_{2},$$

which establishes Equation (54). This bound holds under the assumption $\theta_{uv,t}^{r}\leq 1$ for all neighboring node pairs. This inequality demonstrates that economically modulated attention aggregation does not unboundedly amplify neighboring information when the relation-value transformations remain bounded. In the present task, trade, FDI, patent collaboration, and supply-chain relationships can be used to constrain the cross-national propagation of policy and technological signals. The effective temporal range of historical relationships can also be dynamically adjusted according to the capital environment. Long-term graph memory retains the cumulative effects of industrial construction and reduces the influence of short-term market fluctuations. Consequently, the accuracy and temporal interpretability of policy-diffusion, investment-change, patent-activity, infrastructure-construction, and cross-national industrial-link predictions can be improved.

## 4. Results and Discussion

### 4.1. Experimental Configuration

#### 4.1.1. Hardware and Software Platform

All experiments were conducted on a unified high-performance computing platform. The hardware platform was equipped with dual Intel Xeon Gold 6338 processors operating at a base frequency of 2.00 GHz, providing a total of 64 physical cores, together with 256 GB of DDR4 memory and a 4 TB NVMe solid-state drive. Model training was performed using two NVIDIA RTX 4090 GPUs, each equipped with 24 GB of graphics memory. Data parallelism was employed to accelerate the training of the multimodal model. This computational platform provided sufficient capacity for the simultaneous processing of multilingual policy documents, multitemporal remote-sensing imagery, heterogeneous patent graphs, and economic time series. To reduce the influence of disk input/output operations on training efficiency, the preprocessed text embeddings, remote-sensing image patches, graph-structured data, and economic sequences were stored on the high-speed solid-state drive. Multiprocess data loading was further employed during training to improve data throughput and GPU utilization. The dataset was partitioned chronologically to prevent information leakage from future temporal windows. The training, validation, and test sets accounted for 70%, 15%, and 15% of the complete dataset, respectively. The training set was used for parameter optimization, the validation set was used for hyperparameter selection and early-stopping decisions, and the test set was reserved exclusively for final performance evaluation. Within the temporal range covered by the training and validation sets, blocked five-fold cross-validation was further conducted. In each iteration, four folds were used for model training and the remaining fold was used for validation. This procedure was repeated five times, and the mean and standard deviation of the resulting performance values were reported. For cross-country and cross-industry experiments, data associated with the target test country or industry were completely excluded from model training. This partitioning strategy enabled model generalization to be evaluated under previously unseen regional and industrial conditions. For the asynchronous cross-modal alignment module, the temperature coefficient τ used in contrastive learning was set to 0.07. The candidate lag window between policy and economic signals was defined as 3 time steps before and after the policy event. The corresponding windows for policy–patent and policy–remote-sensing construction signals were set to 6 and 12 time steps before and after the policy event, respectively. During training, the random missing probability $p_{m}$ for an individual modality was set to 0.2, while the probability of jointly masking multiple modalities was set to 0.1. The removal probability for low-weight edges in the graph structure was set to 0.15. In the overall objective function, the weights assigned to the classification loss, regression loss, cross-modal alignment loss, graph-relation prediction loss, modality-reconstruction loss, and reliability-calibration loss were set to 1.0, 1.0, 0.5, 0.5, 0.2, and 0.2, respectively. All experiments were independently repeated using five different random seeds to reduce the influence of random initialization and sample partitioning on the reported results. The proposed framework was implemented using PyTorch 2.11.0 and trained on an NVIDIA RTX 4090 GPU. Unless otherwise specified, the hidden representation dimension was set to 256 throughout the network. The graph memory module employed three graph-attention layers with four attention heads per layer. A temporal window of 12 months was adopted for asynchronous temporal alignment. The model was trained with a batch size of 32 using the AdamW optimizer with an initial learning rate of $1\times 10^{-4}$. Cosine annealing was employed for learning-rate scheduling, with a maximum of 200 training epochs and an early-stopping strategy based on validation performance. The multitask objective was optimized through a weighted combination of individual loss terms, where the corresponding regularization coefficients were selected according to validation-set performance. To facilitate reproducibility, the complete source code, trained model checkpoints, configuration files, and data preprocessing scripts will be publicly released upon acceptance of this work.

#### 4.1.2. Baseline Models and Evaluation Metrics

The baseline models included XLM-R [60], Swin Transformer [61], TimesNet [62], R-GCN [63], Early Fusion [64], Late Fusion [64], Multimodal Transformer [65], HGT [66], and TGN [67]. XLM-R is a multilingual pretrained language model capable of learning unified semantic representations from policy documents written in different languages and was selected because of its strong cross-lingual transfer capability. Swin Transformer extracts local and global features from remote-sensing imagery through hierarchical shifted-window attention and provides relatively high computational efficiency. TimesNet models temporal variation through a two-dimensional representation and can capture both short-term fluctuations and long-term periodic patterns in economic time series. R-GCN employs relation-specific parameters to propagate graph information and is suitable for modeling multirelational networks involving patents, institutions, enterprises, and countries. Early Fusion directly concatenates representations from different modalities at the feature level and provides a structurally simple multimodal baseline. Late Fusion independently generates predictions from each modality before combining the resulting outputs, thereby preserving a relatively high degree of modality independence. Multimodal Transformer applies attention mechanisms to learn interactions among policy text, remote-sensing imagery, patent relations, and economic data and can capture complex cross-modal dependencies. HGT performs heterogeneous attention computation according to node and relation types and is suitable for modeling complex networks of industrial entities. TGN updates node states in dynamic graphs through temporal encoding and memory mechanisms and can characterize the temporal evolution of industrial relationships. The classification metrics included Accuracy, Precision, Recall, F1-score, and ROC-AUC, whereas the regression metrics included MAE, RMSE, MAPE, $R^{2}$, and Pearson’s *r*.

(56) $$\mathrm{Accuracy}=\frac{TP+TN}{TP+TN+FP+FN},\;\mathrm{Precision}=\frac{TP}{TP+FP},\;\mathrm{Recall}=\frac{TP}{TP+FN},$$

(57) $$F1=\frac{2\mathrm{Precision}\times \mathrm{Recall}}{\mathrm{Precision}+\mathrm{Recall}},\;\mathrm{AUC}=\int_{0}^{1}\mathrm{TPR},d\left(\mathrm{FPR}\right),$$

(58) $$\mathrm{MAE}=\frac{1}{n}\sum_{i=1}^{n}\left|y_{i}-{\hat{y}}_{i}\right|,\;\mathrm{RMSE}=\sqrt{\frac{1}{n}\sum_{i=1}^{n}{\left(y_{i}-{\hat{y}}_{i}\right)}^{2}},$$

(59) $$\mathrm{MAPE}=\frac{100\%}{n}\sum_{i=1}^{n}\left|\frac{y_{i}-{\hat{y}}_{i}}{y_{i}}\right|,\;R^{2}=1-\frac{\sum_{i=1}^{n}{\left(y_{i}-{\hat{y}}_{i}\right)}^{2}}{\sum_{i=1}^{n}{\left(y_{i}-\bar{y}\right)}^{2}}.$$

(60) $$r=\frac{\sum_{i=1}^{n}\left(y_{i}-\bar{y}\right)\left({\hat{y}}_{i}-\bar{\hat{y}}\right)}{\sqrt{\sum_{i=1}^{n}{\left(y_{i}-\bar{y}\right)}^{2}\sum_{i=1}^{n}{\left({\hat{y}}_{i}-\bar{\hat{y}}\right)}^{2}}}.$$

Here, TP, TN, FP, and FN denote the numbers of true positives, true negatives, false positives, and false negatives, respectively. The number of samples is denoted by *n*, while $y_{i}$ and ${\hat{y}}_{i}$ represent the ground-truth and predicted values of the *i*-th sample, respectively. Their corresponding means are denoted by $\bar{y}$ and $\bar{\hat{y}}$. The true-positive rate and false-positive rate are represented by TPR and FPR, respectively.

### 4.2. Next-Window AI Computing Infrastructure Construction Event Prediction

This experiment was designed to evaluate the capability of different unimodal, multimodal fusion, and dynamic graph modeling methods to predict artificial intelligence computing infrastructure construction events in the subsequent temporal window. Particular attention was given to whether potential construction activities could be identified in advance from policy, remote-sensing, patent, and economic signals.

**Figure 4:**
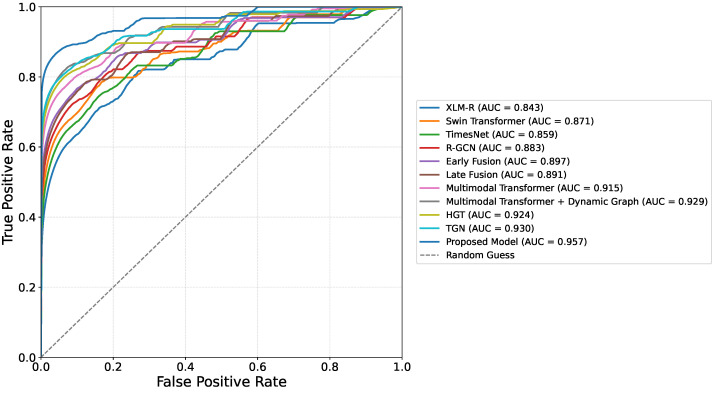
ROC curves of different models for next-window AI computing infrastructure construction event prediction, showing that the proposed model achieves higher true-positive rates and stronger discriminative capability across most decision thresholds.

As shown in Table 3 and Figure 4, the unimodal models exhibited relatively limited performance. XLM-R achieved an Accuracy of 78.64%, an F1-score of 77.37%, and a ROC-AUC of 84.26%. These results indicate that policy documents can capture planning intentions but provide insufficient evidence to determine whether a project has entered the physical construction stage. Swin Transformer achieved an Accuracy of 81.35%, outperforming both XLM-R and TimesNet. This result demonstrates that land-surface hardening, building expansion, and nighttime light variation in remote-sensing imagery provide relatively direct evidence of construction activity. TimesNet achieved an Accuracy of 80.18% and an F1-score of 79.28%. Although periodic variations in investment, trade, and market indicators could be captured, economic fluctuations were not uniquely associated with specific infrastructure projects, which restricted the predictive performance of the model. R-GCN achieved an Accuracy of 82.46% and a ROC-AUC of 88.34%, indicating that multirelational structures among patents, enterprises, institutions, and countries provide useful information regarding industrial agglomeration and technological diffusion. The Accuracy values of Early Fusion and Late Fusion increased to 84.17% and 83.54%, respectively, confirming that complementary information from multiple sources improves prediction performance. However, Early Fusion remained sensitive to differences in feature scale and temporal misalignment, whereas Late Fusion could not sufficiently capture deep cross-modal interactions because integration was performed only at the decision level. The Multimodal Transformer + Dynamic Graph further improved the Accuracy, F1-score, and ROC-AUC to 88.34%, 87.33%, and 92.92%, respectively, demonstrating that incorporating dynamic graph reasoning on top of multimodal feature fusion effectively captures evolving relational dependencies among countries, enterprises, patents, and industrial parks. Compared with the vanilla Multimodal Transformer, these improvements indicate that explicit modeling of heterogeneous graph structures provides complementary information beyond feature-level cross-modal attention. However, this baseline remained consistently inferior to the proposed model across all evaluation metrics. Since it adopts conventional synchronous cross-modal attention without the proposed asynchronous cross-modal alignment module, observations from different modalities are fused according to identical temporal positions, making it difficult to account for heterogeneous response delays among policy announcements, capital investment, patent growth, and physical construction. The comparison therefore demonstrates that the additional performance gain of the proposed framework originates not only from multimodal information fusion and dynamic graph modeling, but also from explicitly modeling cross-modal temporal lag through the proposed asynchronous alignment mechanism. The Multimodal Transformer further increased Accuracy and F1-score to 86.29% and 85.43%, respectively. Its attention-based global interaction mechanism enabled conditional dependencies among policy, image, patent, and economic signals to be learned. Nevertheless, conventional attention generally assumes direct correspondence among modalities at identical temporal positions and therefore cannot adequately model the lagged effects among policy announcements, capital investment, patent growth, and physical construction. HGT achieved an Accuracy of 87.48% and a ROC-AUC of 92.37%, demonstrating that independent transformation parameters for different node and relation types facilitate the modeling of heterogeneous propagation patterns among enterprises, patents, industrial parks, and countries. TGN was the strongest baseline, achieving an Accuracy of 88.16%, an F1-score of 87.30%, and a ROC-AUC of 93.05%. This performance was primarily attributable to its temporal encoding and memory-updating mechanisms, through which historical industrial states and time-varying relations could be retained. The proposed model achieved the best performance across all metrics, with Accuracy, Precision, Recall, F1-score, and ROC-AUC values of 91.72%, 91.08%, 90.64%, 90.86%, and 95.67%, respectively. The narrowest confidence intervals were also obtained, indicating superior stability. From the perspective of model design, asynchronous cross-modal alignment enables independent temporal offsets to be learned for different modalities. Reliability-aware fusion dynamically adjusts modality weights according to cloud coverage, text completeness, patent disclosure delay, and missing economic observations. Economically modulated dynamic graph propagation further constrains the diffusion range of industrial signals across countries, enterprises, and industrial parks. Collectively, these mechanisms reduce the errors introduced by forced synchronization, low-quality modality interference, and static relation assumptions, thereby enabling more accurate identification of computing infrastructure construction events in the subsequent temporal window.

### 4.3. Infrastructure Construction Intensity Forecasting

This experiment was designed to evaluate the capability of different models to forecast continuous changes in artificial intelligence computing infrastructure construction intensity. The complementary contributions of policy, remote-sensing, patent, and economic signals to the characterization of construction scale, expansion rate, and regional heterogeneity were also examined.

**Figure 5:**
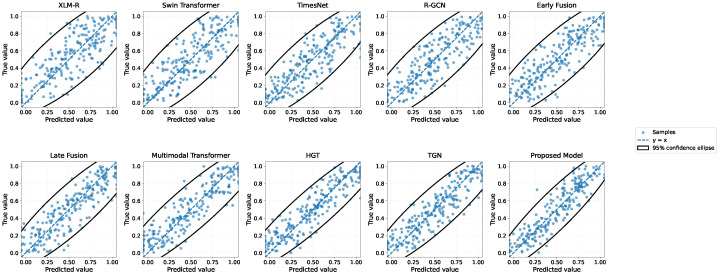
Distributions of predicted and ground-truth infrastructure construction intensity values for different models, with the proposed model exhibiting closer concentration around the ideal regression line and a more compact confidence region.

As shown in Table 4 and Figure 5, among the unimodal models, XLM-R obtained an MAE of 0.184, an RMSE of 0.247, and an $R^{2}$ of only 0.683. These results indicate that policy documents can reflect construction intentions, whereas a substantial temporal lag remains between policy statements and actual construction intensity. Swin Transformer reduced the MAE to 0.162, demonstrating that land-surface hardening, building expansion, and nighttime light variation can be directly captured from remote-sensing imagery. TimesNet further achieved an MAE of 0.155 and an $R^{2}$ of 0.741, indicating that its two-dimensional periodic modeling mechanism is effective in identifying multiscale fluctuations in investment and industrial indicators. R-GCN achieved an MAE of 0.149, outperforming the preceding unimodal models and demonstrating that relational structures among patents, enterprises, and countries provide supplementary information regarding industrial expansion. Early Fusion and Late Fusion achieved MAE values of 0.141 and 0.145, respectively. Although stable improvements were obtained through multimodal information, direct feature concatenation remained insensitive to modality-scale differences, while decision-level fusion could not adequately represent deep cross-modal interactions. The Multimodal Transformer reduced MAE and MAPE to 0.132 and 13.81%, respectively, indicating that attention mechanisms can learn content-dependent cross-modal relationships. HGT further achieved an MAE of 0.127 and an $R^{2}$ of 0.808. Its use of independent projections for different node and relation types enabled heterogeneous propagation among enterprises, patents, industrial parks, and countries to be modeled more accurately. TGN was the strongest baseline, achieving an MAE of 0.122 and a Pearson correlation coefficient of 0.907. These results demonstrate that temporal encoding and memory updating effectively preserve the historical accumulation of construction activities. The proposed model achieved an MAE of 0.104, an RMSE of 0.153, a MAPE of 10.91%, an $R^{2}$ of 0.854, and a Pearson correlation coefficient of 0.928, while also producing the narrowest confidence intervals. From the perspective of its mathematical mechanisms, asynchronous alignment applies learnable temporal offsets to account for different lags among policy announcements, capital investment, and physical construction. Reliability-aware weighting dynamically suppresses low-quality modalities according to observation uncertainty. Economically modulated dynamic graph propagation uses nonnegative normalized weights to limit the amplification of neighboring noise, while long-term memory accumulates persistent industrial construction states. Consequently, both absolute errors caused by local anomalies and deviations around construction-intensity peaks are reduced, allowing the overall temporal trend of infrastructure construction intensity to be recovered more accurately.

### 4.4. Ablation Study of the Proposed Model

This ablation experiment was designed to evaluate the independent contributions of asynchronous cross-modal alignment, reliability-aware fusion, and economically modulated dynamic heterogeneous graph evolution. The effects of these mechanisms on the prediction of artificial intelligence computing infrastructure construction events in the subsequent temporal window were examined.

**Figure 6:**
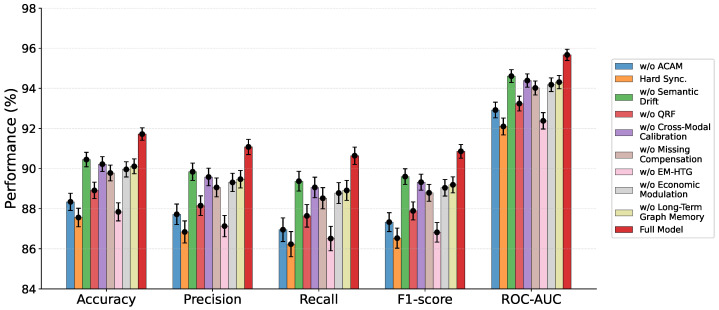
Performance comparison of different ablation configurations in terms of Accuracy, Precision, Recall, F1-score, and ROC-AUC, demonstrating that the full model achieves the best and most stable results across all evaluation metrics.

As shown in Table 5 and Figure 6, the full model achieved the best overall performance, with an Accuracy of 91.72%, an F1-score of 90.86%, and a ROC-AUC of 95.67%. When ACAM was removed, Accuracy decreased to 88.34%, indicating that temporal misalignment among policy, patent, economic, and remote-sensing signals substantially affects predictive performance. Accuracy further decreased to 87.56% under hard synchronous alignment, demonstrating that forced matching within the same temporal window can incorrectly associate policy announcements with physical construction activities that have not yet occurred. After removal of the semantic drift constraint, an Accuracy of 90.45% was retained, although all metrics remained below those of the full model. This finding indicates that the constraint primarily stabilizes cross-modal semantic mappings and prevents learned temporal offsets from disrupting industrial-topic consistency. When RAMF was removed, Accuracy decreased to 88.91%, demonstrating that fixed fusion or fusion without explicit quality assessment is vulnerable to cloud contamination, incomplete policy documents, delayed patent disclosure, and missing economic observations. The removal of cross-modal consistency calibration and missing-modality compensation reduced Accuracy to 90.22% and 89.78%, respectively. These results indicate that consistency information facilitates the detection of anomalous modalities, whereas the reconstruction mechanism maintains prediction continuity under incomplete observations. When EM-HTG was removed, Accuracy and F1-score decreased to 87.84% and 86.82%, respectively, representing one of the largest performance reductions among the principal modules. This result demonstrates that dynamic relations among countries, enterprises, patents, and industrial parks are essential for construction-event identification. The removal of economic modulation reduced Accuracy to 89.96%, indicating that capital flows, market pressure, and investment trends effectively regulate the propagation intensity of industrial relations. When long-term graph memory was removed, Accuracy decreased to 90.11%, confirming that infrastructure construction exhibits persistent cumulative characteristics that cannot be adequately represented using only the current temporal window. From the perspective of mathematical properties, ACAM distributes matching probabilities across multiple candidate windows through continuous learnable temporal offsets and normalized soft attention, thereby reducing the information loss associated with discrete hard alignment. RAMF applies bounded reliability weights and cross-modal distance calibration to adaptively attenuate highly uncertain modalities. EM-HTG combines relation-specific parameters, nonnegative attention weights, economic gating, and recurrent memory to preserve long-term industrial evolution while controlling noise propagation. The complementary effects of these components explain why the full model achieved not only the highest predictive accuracy but also the narrowest confidence intervals and the most stable cross-modal decision performance.

### 4.5. Qualitative Analysis of ACAM and RAFM

**Figure 7:**
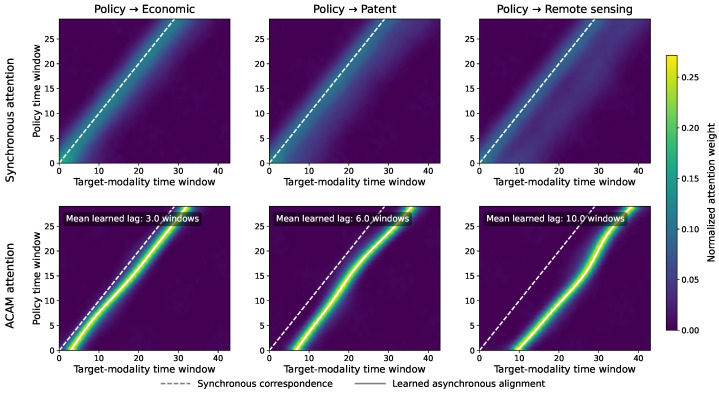
Visualization of representative cross-modal attention learned by the proposed ACAM.

ACAM. To further interpret the behavior of the proposed ACAM, Figure 7 visualizes representative cross-modal attention maps after temporal-offset correction. Unlike conventional synchronous attention, the highest attention responses are consistently shifted away from the main diagonal, indicating that the model does not simply associate observations from identical timestamps. Instead, it automatically aligns policy events with subsequent patent activities, economic responses, and remote-sensing observations according to their learned temporal dependencies. Moreover, the attention distributions remain locally concentrated rather than randomly scattered, demonstrating that the learned temporal offsets are stable and semantically meaningful across different industrial development stages. These observations suggest that ACAM effectively captures asynchronous cross-modal evolution and establishes reliable semantic correspondences between heterogeneous modalities. The qualitative evidence is consistent with the quantitative improvements reported in previous sections and provides an intuitive explanation for the superior predictive performance of the proposed framework.

**Figure 8:**
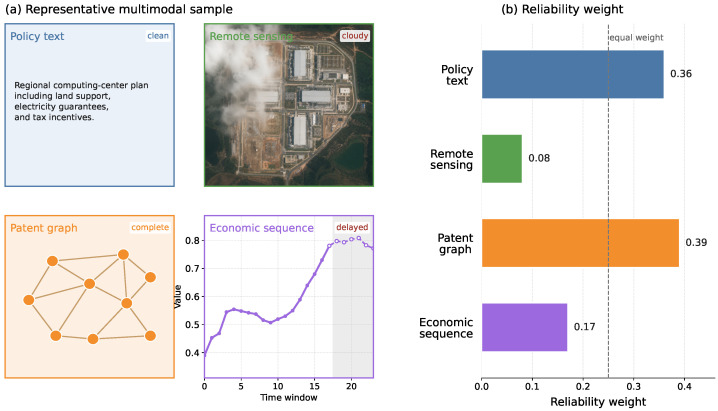
Representative case illustrating the RAMF process.

RAFM. Figure 8 presents a representative inference case of the proposed Reliability-aware Fusion module. In this example, the remote-sensing modality is partially contaminated by cloud cover, while the economic sequence contains delayed observations. Accordingly, the reliability estimator assigns substantially lower fusion weights to these unreliable modalities and places greater emphasis on policy documents and patent information, both of which remain complete and semantically consistent. Consequently, the fused representation is dominated by high-quality information sources rather than noisy observations. This example demonstrates that the proposed reliability-aware fusion mechanism adaptively suppresses unreliable modalities instead of treating all inputs equally, thereby improving robustness under realistic industrial monitoring conditions.

### 4.6. Discussion

The results demonstrate that policy documents, remote-sensing imagery, patent activity, and economic signals do not evolve synchronously. Instead, their effects are gradually transmitted through a pathway involving policy announcement, capital investment, technological agglomeration, and physical infrastructure construction. This characteristic has direct practical relevance to the construction of artificial intelligence computing centers, semiconductor manufacturing facilities, and industrial parks. For example, after computing subsidies, land incentives, and energy-support policies are introduced in a region, increases in corporate financing, fixed-asset investment, and equipment imports may first be observed in the short term. These changes may subsequently be followed by increases in patents and collaborative relationships related to chips, servers, cooling systems, and data centers. Only at a later stage may land-surface hardening, factory expansion, enhanced nighttime illumination, and thermal emissions become visible in remote-sensing imagery. These temporally lagged and sequential signals can be identified by the proposed model, enabling the transition of a project into substantive construction to be assessed in advance and the intensity of construction in the subsequent stage to be forecast. In governmental industrial management, the proposed model can be applied to monitor the implementation of announced development plans and identify regions in which strong policy support has not resulted in a corresponding construction response. Such information can support adjustments to fiscal subsidies, land allocation, electricity guarantees, and project-approval schedules. In industrial-park management, remote-sensing construction changes, enterprise patent collaboration, and capital activity can be jointly analyzed to estimate industrial-park expansion, the establishment of computing centers, and demand for supporting infrastructure. These predictions can support the planning of substations, communication networks, roads, and cooling-water systems. In enterprise site selection and supply-chain management, equipment manufacturers, semiconductor companies, and cloud-service providers can use the predicted results to assess future computing demand and construction speed across regions, thereby facilitating advance planning for the production and inventory of servers, network equipment, transformers, and cooling systems. Cross-country comparisons can also be used to identify policy diffusion, investment relocation, and industrial-chain restructuring, providing temporally interpretable evidence for artificial intelligence infrastructure planning and semiconductor industrial deployment.

### 4.7. Limitations and Future Work

Several limitations remain. First, the policy documents, remote-sensing images, patent relations, and economic indicators were primarily obtained from publicly accessible databases. Differences in information disclosure systems, statistical definitions, patent publication cycles, and remote-sensing image quality across countries may result in uneven regional data density and may affect the comparability of cross-country predictions. Second, infrastructure construction events and construction intensity were mainly annotated using publicly available project information, remote-sensing changes, and industrial records. Consequently, confidential projects, underground facilities, and internal modifications to existing buildings may not have been fully identified. Third, the current model primarily characterizes temporal relationships among policy, capital, technological activity, and infrastructure construction, whereas factors such as land approval, electricity capacity, water-resource constraints, construction permits, and local implementation capacity have not yet been fully incorporated. Future research will therefore introduce more direct observations, including power-grid load, energy consumption, construction permits, equipment procurement, and industrial-park operation records, while unified cross-country data calibration and event-annotation standards will be established. The study scope can also be extended to additional developing countries and emerging computing markets, and long-term rolling validation and real-project tracking can be conducted to assess model stability under different policy systems, industrial foundations, and missing-data conditions. By further incorporating policy-scenario simulation and causal analysis, correlations between policy announcements and physical construction may be distinguished from genuine causal pathways, thereby increasing the practical value of the resulting predictions. Although the proposed framework demonstrates promising performance, several practical limitations should be acknowledged. First, the policy modality primarily relies on publicly available policy documents, which may not fully capture unpublished regulations or newly issued policy information. Second, the quality of remote-sensing imagery may vary across geographical regions because of differences in spatial resolution, observation frequency, cloud contamination, and acquisition conditions, potentially affecting the robustness of cross-regional deployment. In addition, economic statistics are often subject to reporting delays and subsequent revisions, causing temporal discrepancies between published indicators and actual industrial conditions. Future research will investigate the integration of real-time policy information, higher-quality remote-sensing observations, multiple economic data sources, and uncertainty-aware learning strategies to further improve the robustness, generalization capability, and practical applicability of the proposed framework in real-world scenarios. In addition to predictive performance, computational efficiency and scalability are important considerations for practical deployment. Since the primary objective of this work is to develop and validate a unified asynchronous multimodal learning framework, dedicated evaluations of GPU memory consumption, inference time, and scalability under different graph sizes are beyond the scope of the current study. From a methodological perspective, the computational cost mainly arises from multimodal feature encoding, heterogeneous graph message passing, asynchronous temporal alignment, and reliability-aware multimodal fusion, and is jointly influenced by the complexity of neural encoders, the size of the heterogeneous graph, and the frequency of cross-modal interactions. Future work will perform more comprehensive efficiency evaluations on larger industrial datasets, including GPU memory usage, inference latency, and scalability analyses under different graph scales. In addition, optimization strategies such as graph sampling, mini-batch graph processing, dynamic graph compression, and distributed computation will be investigated to further improve the deployment efficiency and scalability of the proposed framework in real-world industrial applications.

## 5. Conclusions

To address the difficulty of synchronously observing policy intentions, capital activities, technological innovation, and physical construction during the rapid expansion of artificial intelligence computing infrastructure and the semiconductor industry, a multimodal industrial sensing dataset comprising policy documents, remote-sensing imagery, patent relations, and economic time series was constructed. An artificial intelligence-driven sensing framework was further developed for infrastructure construction event identification and construction intensity forecasting. Differential temporal lags among policy announcements, capital investment, patent growth, and physical construction are learned through an asynchronous cross-modal alignment mechanism. Modality contributions are dynamically adjusted through reliability-aware fusion according to text completeness, cloud coverage in remote-sensing imagery, patent disclosure delays, and missing economic observations. Time-varying propagation relationships among countries, enterprises, patents, and industrial parks are modeled using an economically modulated dynamic heterogeneous graph. Experimental results demonstrate that the proposed model achieved an Accuracy of 91.72%, a Precision of 91.08%, a Recall of 90.64%, an F1-score of 90.86%, and a ROC-AUC of 95.67% in next-window construction event prediction, outperforming baseline methods including XLM-R, Swin Transformer, TimesNet, HGT, and TGN. In infrastructure construction intensity forecasting, an MAE of 0.104, an RMSE of 0.153, a MAPE of 10.91%, an $R^{2}$ of 0.854, and a Pearson correlation coefficient of 0.928 were obtained. Ablation experiments further confirmed the effectiveness of asynchronous alignment, reliability calibration, missing-modality compensation, economic modulation, and long-term graph memory. These findings indicate that multisource sensing information can be transformed into temporally interpretable representations of industrial states, thereby providing reliable technical support for computing center site selection, industrial park expansion, power and communication infrastructure allocation, equipment supply planning, and policy implementation assessment.

## Figures and Tables

**Table 1:** Collection scale of the multimodal industrial evolution dataset.

Data Type	Sensor Model	Quantity
Policy text data	PTX-01 Multilingual Policy Text Sensor	46,280 documents
Remote-sensing imagery	Sentinel-2 MSI, Landsat 8/9 OLI/TIRS, and VIIRS DNB	18,640 scenes
Patent relation data	PGX-02 Patent Graph Sensor	1,342,760 records
Economic time-series data	ETS-03 Economic Time-Series Sensor	312,480 observations

**Table 2:** Notation used in manuscript.

Symbol	Description
*m*	Modality index
$x^{m}$	Original sample of modality *m*
${\tilde{x}}^{m}$	Augmented sample of modality *m*
$T_{m}\left(\cdot \right)$	Augmentation operator for modality *m*
$\theta_{m}$	Augmentation parameters
$L_{\mathrm{text}}$	Semantic consistency loss for policy-text augmentation
$I_{t}$	Remote-sensing image at time step *t*
${\tilde{I}}_{t}$	Augmented remote-sensing image
G(·)	Geometric image transformation
$M_{t}$	Image mask
$A^{r}$	Original adjacency matrix of relation type *r*
${\tilde{A}}^{r}$	Augmented adjacency matrix
$R^{r}$	Reconstructed edge matrix
$x_{t}$	Original economic time-series observation
${\tilde{x}}_{t}$	Augmented economic time-series observation
${\hat{x}}_{t}$	Interpolated economic observation
$z_{m}$	Modality availability mask
$p_{m}$	Missing probability of modality *m*
$e_{\mathrm{miss}}^{m}$	Learnable missing-modality token
P	Policy feature sequence
V	Remote-sensing feature tensor
G	Patent graph feature matrix
E	Economic feature sequence
$L_{p},L_{v},L_{e}$	Lengths of the policy, visual, and economic sequences
$H_{v},W_{v},C_{v}$	Height, width, and channel dimension of the visual feature map
$N_{g}$	Number of nodes in the patent graph
$\alpha_{t,h,w}^{v}$	Spatial attention weight
$v_{t}$	Spatially pooled visual representation
${\hat{Z}}_{m}$	Modality representation in the shared semantic space
$e_{m}$	Modality-type embedding
$\delta_{ij}^{a\to b}$	Predicted temporal offset
ρ(·)	Relative temporal encoding function
${\tilde{\Delta }}_{ij}^{a\to b}$	Corrected temporal distance
$s_{ij,r}^{a\to b}$	Cross-modal attention score
$A_{ij,r}^{a\to b}$	Asynchronous attention weight
$g_{i}^{a\leftarrow b}$	Cross-modal gating weight
$h_{i}^{\mathrm{align}}$	Aligned multimodal representation
$L_{\mathrm{temporal}}$	Temporal regularization loss
$\eta_{1},\eta_{2}$	Loss weighting coefficients
$X^{m}$	Aligned feature representation of modality *m*
$H_{m},W_{m},C_{m}$	Spatial/temporal dimensions and channel width of modality *m*
$F^{m}$	Scale-adapted feature representation
$H_{f},W_{f},C_{f}$	Unified feature dimensions after scale adaptation
$P_{m}\left(\cdot \right)$	Scale-adaptation network
$q^{m}$	Modality quality descriptor
$c^{m}$	Data completeness
$o^{m}$	Observation quality
$f^{m}$	Temporal freshness
$u^{m}$	Predictive uncertainty
$s^{m}$	Alignment stability
$\rho^{m}$	Modality reliability score
$g^{m}$	Global feature representation of modality *m*
$d^{m,n}$	Cross-modal feature distance
$\chi^{m}$	Cross-modal consistency coefficient
$\omega^{m}$	Adaptive fusion weight
${\hat{F}}^{m}$	Reconstructed feature of missing modality *m*
$D_{m}\left(\cdot \right)$	Missing-modality reconstruction network
${\bar{F}}^{m}$	Feature used for multimodal fusion
F(·)	Multimodal feature fusion operator
$G_{t}$	Dynamic heterogeneous graph at time step *t*
Q	Set of node types
R	Set of relation types
$X_{t}^{q}$	Feature matrix of node type *q*
$N_{q},C_{q}$	Number of nodes and feature dimension of node type *q*
$A_{t}^{r}$	Adjacency matrix of relation type *r*
$\gamma_{uv,t}^{r}$	Economic modulation coefficient
$\phi_{uv,t}^{r}$	Multi-scale temporal basis functions
$\beta_{uv,t}^{r}$	Temporal-scale mixture coefficients
$\vartheta_{uv,t}^{r}$	Economically modulated temporal weight
$\alpha_{uv,t}^{r,l,k}$	Relation-aware attention weight
$m_{v,t}^{l,k}$	Aggregated relational message
$H_{v,t}^{l}$	Node representation at graph layer *l*
$M_{v,t}$	Long-term graph memory
$i_{v,t}$	Memory input gate
$f_{v,t}$	Memory retention gate
$Y_{v,t}$	Final node representation
$\Pi_{q}$	Permutation matrix for node type *q*
$L_{g}$	Number of graph evolution layers
$C_{M}$	Channel dimension of the graph memory

**Table 3:** Performance comparison of different methods for next-window AI computing infrastructure construction event prediction. Results are reported as the mean ± 95% confidence interval over five independent runs. This baseline is identical to the ablation model without the asynchronous cross-modal alignment module (w/o ACAM). ↑: Higher values indicate better performance. underline indicates the second best. **Bold** indicates optimal performance.

Method	Accuracy (%) ↑	Precision (%) ↑	Recall (%) ↑	F1-Score (%) ↑	ROC-AUC (%) ↑
XLM-R	78.64±0.71	77.92±0.83	76.85±0.92	77.37±0.74	84.26±0.65
Swin Transformer	81.35±0.63	80.72±0.76	79.94±0.81	80.33±0.69	87.08±0.58
TimesNet	80.18±0.68	79.65±0.79	78.92±0.86	79.28±0.72	85.91±0.61
R-GCN	82.46±0.57	81.88±0.69	81.15±0.75	81.51±0.62	88.34±0.54
Early Fusion	84.17±0.52	83.62±0.63	82.95±0.70	83.28±0.58	89.73±0.49
Late Fusion	83.54±0.55	82.96±0.67	82.41±0.72	82.68±0.61	89.12±0.51
Multimodal Transformer	86.29±0.46	85.74±0.55	85.12±0.62	85.43±0.49	91.46±0.43
Multimodal Transformer + Dynamic Graph	88.34±0.43	87.72±0.51	86.95±0.59	87.33±0.47	92.92±0.39
HGT	87.48±0.42	86.95±0.51	86.38±0.57	86.66±0.45	92.37±0.39
TGN	88.16±0.37	87.52±0.46	87.08±0.52	87.30±0.41	93.05±0.35
Proposed Model	91.72±0.31 ^∗^	91.08±0.38 ^∗^	90.64±0.43 ^∗^	90.86±0.34 ^∗^	95.67±0.28 ^∗^

^∗^ indicates statistically significant improvement over the strongest baseline (TGN) (p<0.05).

**Table 4:** Performance comparison of different methods for infrastructure construction intensity forecasting. Results are reported as the mean ± 95% confidence interval over five independent runs. ↑: Higher values indicate better performance; ↓: Lower values indicate better performance. underline indicates the second best. **Bold** indicates optimal performance.

Method	MAE ↓	RMSE ↓	MAPE (%) ↓	$R^{2}$↑	Pearson *r* ↑
XLM-R	0.184±0.006	0.247±0.008	18.92±0.73	0.683±0.014	0.832±0.011
Swin Transformer	0.162±0.005	0.221±0.007	16.74±0.65	0.724±0.012	0.851±0.010
TimesNet	0.155±0.005	0.214±0.007	15.96±0.61	0.741±0.011	0.861±0.009
R-GCN	0.149±0.004	0.207±0.006	15.32±0.57	0.756±0.010	0.870±0.008
Early Fusion	0.141±0.004	0.198±0.006	14.67±0.52	0.773±0.009	0.881±0.008
Late Fusion	0.145±0.004	0.203±0.006	14.95±0.55	0.766±0.010	0.876±0.008
Multimodal Transformer	0.132±0.003	0.188±0.005	13.81±0.46	0.792±0.008	0.892±0.007
HGT	0.127±0.003	0.181±0.005	13.24±0.42	0.808±0.008	0.901±0.006
TGN	0.122±0.003	0.176±0.004	12.87±0.39	0.817±0.007	0.907±0.006
Proposed Model	0.104±0.002 ^∗^	0.153±0.004 ^∗^	10.91±0.34 ^∗^	0.854±0.006 ^∗^	0.928±0.005 ^∗^

^∗^ indicates statistically significant improvement over the strongest baseline (TGN) (p<0.05).

**Table 5:** Ablation study of the proposed model on AI computing infrastructure construction event prediction. Results are reported as the mean ± 95% confidence interval over five independent runs. ↑: Higher values indicate better performance. underline indicates the second best. **Bold** indicates optimal performance.

Model Configuration	Accuracy (%) ↑	Precision (%) ↑	Recall (%) ↑	F1-Score (%) ↑	ROC-AUC (%) ↑
w/o ACAM	88.34±0.43	87.72±0.51	86.95±0.59	87.33±0.47	92.92±0.39
Hard Synchronous Alignment	87.56±0.46	86.84±0.55	86.23±0.63	86.53±0.50	92.10±0.42
w/o Semantic Drift Constraint	90.45±0.36	89.84±0.43	89.37±0.49	89.60±0.39	94.61±0.32
w/o RAMF	88.91±0.41	88.15±0.49	87.64±0.56	87.89±0.45	93.24±0.37
w/o Cross-Modal Consistency Calibration	90.22±0.37	89.58±0.44	89.06±0.51	89.32±0.40	94.39±0.33
w/o Missing-Modality Compensation	89.78±0.39	89.06±0.47	88.52±0.53	88.79±0.42	94.02±0.35
w/o EM-HTG	87.84±0.45	87.13±0.53	86.51±0.61	86.82±0.49	92.38±0.41
w/o Economic Modulation	89.96±0.38	89.31±0.45	88.78±0.52	89.04±0.41	94.18±0.34
w/o Long-Term Graph Memory	90.11±0.37	89.47±0.44	88.91±0.50	89.19±0.40	94.31±0.33
Full Model	91.72±0.31	91.08±0.38	90.64±0.43	90.86±0.34	95.67±0.28

## Data Availability

The data presented in this study are available on request from the corresponding author.
